# Increased risk of death following release from incarceration: an individual participant data meta-analysis of 1,314,568 adults in eight countries.

**DOI:** 10.23889/ijpds.v7i3.1872

**Published:** 2022-08-25

**Authors:** Rohan Borschmann, Claire Keen, Jesse Young, Stuart Kinner

**Affiliations:** 1 University Of Melbourne; 2 Curtin University

## Objectives

People released from incarceration are at increased risk of death from diverse causes. We aimed to calculate the incidence of all-cause and cause-specific death after release from incarceration and identify individual-level risk factors for death.

## Approach

We conducted a series of individual participant data meta-analyses using data from >1.3 million adults released from incarceration in eight countries from 1980-2018. We used random effects meta-analysis to estimate the pooled all-cause and cause-specific crude mortality rates (CMRs), with 95% confidence intervals (CI) for the entire follow-up period, and for specific time periods after release from incarceration, overall and stratified by age, sex, and region.

## Results

We included 1,395,318 people, 10,164,341 person-years of follow-up time, and 72,920 deaths in our analyses. The overall pooled CMR was 727 (95%CI: 623-840) per 100,000 person-years, with no difference between males and females. The risk of death was highest during the first week following release (all-cause CMR: 1,612, 95%CI: 1048-2,287, I2=91.5%), and the three most common causes of death across the entire follow-up period were 1) alcohol and other drug poisoning (CMR=144, 95%CI: 99-197); 2) cardiovascular disease (CMR: 102, 95%CI: 85-121); and 3) cancer and other neoplasms (CMR=74, 95%CI: 85-121). Leading causes of death varied across time periods following release from incarceration.

## Conclusion and Relevance

Our findings indicate the need for routine monitoring of mortality following release from incarceration. The distribution of cause of death varies over time, such that clinical decision-making needs to be informed by the proximity to release from incarceration. The elevated risk of death in first 7 days following release highlights the urgent need for coordinated transitional care – including substance use and mental health treatment – and injury prevention initiatives.

